# The association between IL-17 gene variants and risk of colorectal cancer in a Chinese population: A case–control study

**DOI:** 10.1042/BSR20190013

**Published:** 2019-11-26

**Authors:** Haiyang Feng, Rongbiao Ying, Tengjiao Chai, Hailang Chen, Haixing Ju

**Affiliations:** 1 Department of Colorectal Surgery, Institute of Cancer Research and Basic Medical Sciences of Chinese Academy of Sciences, Cancer Hospital of University of Chinese Academy of Sciences, Zhejiang Cancer Hospital, No. 1, East Banshan Road, Gongshu District, Hangzhou, Zhejiang, China; 2 Department of Surgical Oncology, Taizhou Cancer Hospital, No. 50, Zhenxin Road, Wenling, Taizhou, Zhejiang, China; 3 Department of Colorectal Surgery, Quzhou Kecheng People’s Hospital, No. 172, Shuanggang Road, Quzhou, Zhejiang, China; 4 Department of Oncology, Lanxi People’s Hospital, No. 1359, Xishan Road, Lanxi, Jinghua, Zhejiang, China

**Keywords:** IL-17, case-control study, colorectal cancer, SNPs

## Abstract

Interleukin (IL)-17 have been reported to be associated with the pathogenesis of colorectal cancer (CRC). Few studies investigated the association between *IL-17* gene polymorphisms and risk of CRC with inconsistent findings. Thus, we recruited 352 CRC cases and 433 controls in a Chinese population and their genotyping was done using polymerase chain reaction-restriction fragment length polymorphism method. Our data showed that *IL-17A* rs2275913 polymorphism was associated with the increased risk of CRC, while no association was observed for *IL-17F* rs763780 polymorphism. Stratified analyses revealed that the significant association was also obtained in the females, smokers, drinkers and age ≥ 60 years groups for rs2275913 polymorphism. Moreover, the CC and/or GC genotype of rs2275913 polymorphism were correlated with TNM stage and lymph node metastasis. No association was shown between *IL-17F* rs763780 polymorphism and clinical characteristics of CRC. In conclusion, our data indicate that *IL-17A* rs2275913 polymorphism but not *IL-17F* rs763780 polymorphism contributes to increased risk for CRC patients in this Chinese population.

## Introduction

Colorectal cancer (CRC) is the third most common cancer and the fourth most common cause of cancer death [1]. More than 1.2 million patients are diagnosed with CRC annually and more than 600,000 die from this disorder [2]. It is well known that environmental and inherited genetic factors made great contributions to susceptibility to CRC [3]. Genome-wide association studies (GWAS) and meta-analysis using 1000 Genomes for imputation have identified novel risk variants for CRC patients [4].

Two inflammatory cytokines, Interleukin (IL)-17A and IL-23, produced by myeloid cells and different lymphocyte subsets, have been implicated in the pathogenesis of inflammatory associated cancers such as CRC [5]. The up-regulation of IL-17 ad IL-23 was observed in a mouse model of colorectal tumorigenesis [6]. In CRC, elevated expression of IL-17A was associated with adverse prognostic outcome and rapid progression to metastatic disease [7]. Th17-type cytokines (IL-17A, IL-17F, IL-21, IL-22), IL-6 and tumor necrosis factor-α synergistically activate STAT3 and NF-κB to promote CRC growth [8]. Single-nucleotide polymorphism (SNP) in IL-17 gene may influence genomic stability and increase the production of IL-17, thereby conferring susceptibility to CRC [9].

Many studies have investigated the association between *IL-17* gene polymorphisms and risk of CRC [9–15], mainly focusing on two polymorphisms (rs2275913, *IL-17A*; rs763780, *IL-17F*). However, their findings were conflicting. Furthermore, there is no study to investigate the association between *IL-17A* rs2275913 polymorphism and risk of CRC in a Chinese population. In addition, only one study [12] has thrown lights on the rs763780 polymorphism for Chinese population. Therefore, we conducted this hospital-based case–control study to evaluate the effects of *IL-17A* rs2275913 and *IL-17F* rs763780 polymorphisms on the risk of CRC.

## Patients and methods

### Subjects

In the present study, 352 CRC patients with newly histopathologically diagnosed CRC and 433 sex- and age-matched controls were recruited from Zhejiang Cancer Hospital, Taizhou Cancer Hospital, Quzhou Kecheng People’s Hospital and Lanxi People’s Hospital from May 2012 to May 2018. No patients had received radiotherapy or chemotherapy prior to surgery. Approximately 58.2% of CRC patients were rectal cancer and were classified according to the American Joint Committee on cancer (AJCC) classification system. The control groups were selected from individuals receiving health examinations at the same period. The individuals with family history of cancer or digestive diseases were excluded.

Data on demographic and risk factor information for all subjects were obtained using a self-designed questionnaire, including body mass index (BMI), smoking status, alcohol consumption and family history of cancer. The individuals who smoked at least one cigarette per day at least one year was defined as “smoker”. Individuals were defined as drinkers if they drank alcohol at least once a week for more than 1 year. Subjects with at least one first-degree relative or two second-degree relatives having CRC were defined as having family history of cancer. We obtained clinical information about C-reactive protein (CRP); erythrocyte sedimentation rate (ESR); TNM stage, localization of tumor, tumor size, differentiation, lymph node metastasis and histopathological characteristics from the medical record. The study was approved by the Ethics Committee of the above four hospitals and met the standards of Declaration of Helsinki. Written informed consent was obtained from each subject.

### Blood sampling and genotyping

Peripheral blood (2 ml) was taken from all subjects and genomic DNA was extracted from peripheral blood using the TIANamp Blood DNA kit (Tiangen Biotech, Beijing, China) according to manufacturer’s instructions. The quality and concentration of extracted DNA was measured in two OD wavelength 260 and 280 nm using NanoDrop (Thermo Scientific, U.S.A.). SNP genotyping was performed using polymerase chain reaction-restriction fragment length polymorphism method. The primers used for the nucleotide extension reaction were AACAAGTAAGAATGAAAAGAGGACATGGT (forward) and CCCCCAATGAGGTCATAGAAGAATC (reverse) for rs2275913 and ACCAAGGCTGCTCTGTTTCT (forward) and GGTAAGGAGTGGCATTTCTA (reverse) for rs763780 polymorphism. For PCR, 25 μl reaction mixture contained as follows: 2.5 μl of 10× reaction buffer (with 1.5 mM MgCl_2_), 2 μl of deoxynucleotide triphosphate (dNTP; 2.5 mM), 2 μl of each pair primer, 50 ng DNA template, 1 μl of 0.4U Taq polymerase (Applied Biosystems, Evry, France) and 14.5 μl ddH_2_O. The cycling program involved preliminary denaturation at 94°C for 5 min, followed by 35 cycles of 94°C for 30 s, 57°C for 30 s, and 72°C for 30 s with a final extension at 72°C for 8 min. The digested PCR products were with XagI (Fermentas, Lithuania) for *IL-17A* rs2275913 polymorphism and NlaIII (BioLabs, England) for *IL-17F* rs763780 polymorphism overnight at 37°C. The PCR products were analyzed by horizontal electrophoresis on an ethidium bromide-stained agarose gel (2% w/v) and then photographed. About 4% of selected samples were reconfirmed with direct sequencing to ensure the genotyping accuracy.

### Statistical analysis

The demographic variables were expressed as means ± standard deviation (continuous variables) and frequencies and percentages (categorical variables) respectively. Differences between means were compared by Student’s *t*-test or Mann–Whitney *U* test (where the data were not distributed normally). The chi-square test was used to evaluate the differences in frequency distributions of categorical between cases and controls. The Hardy–Weinberg equilibrium (HWE) was applied to test for deviation between observed and expected frequencies among controls using a goodness-of-fit chi-square test. The SNP-associated disease risk was assessed with logistic regression analysis adjusted for age and sex. *P* < 0.05 were considered statistically significant. All statistical analyses were conducted using SPSS 22.0 software (SPSS Inc., Chicago, U.S.A.).

## Results

### Characteristics of the study population

The demographic and medical data of participants are shown in Table 1. The mean age of CRC patients was 62.27 years compared with 62.38 years of control groups, which revealed no statistically difference (*P* = 0.848). The distribution of sex, smoking, BMI and drinking did not differ significantly. Among the 352 cases, 215 (61.1%) were adenocarcinoma; 123 (34.9%) were squamous cell carcinoma; and 14 (4.0%) were other types of CRC. We have also investigated other clinical information of CRC patients, including histological grade, TNM stage, tumor size and lymph node metastasis.

**Table 1:** Patient demographics and risk factors in colorectal cancer.

Characteristics	Case (*N* = 352)	Control (*N* = 433)	*P*
Age, years	62.27 ± 8.10	62.38 ± 7.78	0.848
Sex			0.629
Male	69 (19.6%)	79 (18.2%)	
Female	283 (80.4%)	354 (81.8%)	
BMI, kg/m^2^	25.12 ± 4.00	24.84 ± 3.80	0.306
Smoking			0.361
Yes	192 (54.5%)	222 (51.3%)	
No	160 (45.5%)	211 (48.7%)	
Alcohol			0.774
Yes	206 (58.5%)	249 (57.5%)	
No	146 (41.5%)	184 (42.5%)	
CRP, mg/l	7.73 ± 17.38		
ESR, mm/h	10.85 ± 12.00		
Family history			
Yes	47 (13.4%)		
No	305 (86.6%)		
Histological grade			
Well differentiated	34 (9.7%)		
Moderately differentiated	275 (78.1%)		
Poorly differentiated	43 (12.2%)		
TNM stage			
I+II	192 (54.5%)		
III+IV	160 (45.5%)		
Tumor size			
>5 cm	202 (57.4%)		
≤5 cm	150 (42.6%)		
Lymph node metastasis			
No	230 (65.3%)		
Yes	122 (34.7%)		
Histology			
Adenocarcinoma	338 (96.0%)		
Squamous cell carcinoma	10 (2.8%)		
Others	4 (1.2%)		

BMI, body mass index; CRP, C-reactive protein; ESR, erythrocyte sedimentation rate; TNM, tumor node metastasis.

### *IL-17* gene polymorphisms analysis

The product lengths (bp) of different genotypes were as following: AA: 102bp, AG: 102 bp + 68bp + 34bp, GG: 68bp + 34bp (IL-17A rs2275913 polymorphism); TT: 63bp + 80bp, CT: 143bp + 80bp + 63bp, CC: 143bp (IL-17F rs763780 polymorphism). Table 2 shows the genotype and allele distributions for *IL-17* gene polymorphisms in the CRC patients and controls. The observed genotype frequencies for the two polymorphisms (rs2275913 and rs763780) in the controls did not departure from HWE. For rs2275913 polymorphism, A allele was related to increased risk for CRC (AA vs GG: OR, 1.31; 95% CI, 1.06–1.64; *P* = 0.014); the AA genotype (not GA genotype) had a significantly elevated risk for CRC compared to GG genotype (AA vs GG: OR, 1.72; 95% CI, 1.02–2.88; *P* = 0.041). Similarly, AA+GA genotype or A allele was associated with the increased risk for CRC. Further, rs2275913 polymorphism was significant in homozygous, dominant and allelic models after adjusting for sex and age. However, no significant associations with the CRC risk was demonstrated for rs763780 polymorphism before and after adjusting for sex and age.

**Table 2:** Genotype frequencies of IL-17 gene polymorphisms in cases and controls

Models	Genotype	Case (*n*, %)	Control (*n*, %)	OR (95% CI)	*P*-value	*OR (95% CI)	*P-value
rs2275913							
	GG	160 (45.6%)	231 (53.6%)	1.00 (reference)			
Heterozygous	GA	154 (43.9%)	169 (39.2%)	1.31 (0.98–1.76)	0.075	1.32 (0.98–1.77)	0.071
Homozygous	AA	37 (10.5%)	31 (7.2%)	**1.72 (1.02–2.88)**	**0.041**	**1.73 (1.03–2.91)**	**0.039**
Dominant	GG	160 (45.6%)	231 (53.6%)	1.00 (reference)			
	AA+GA	191 (54.4%)	200 (46.4%)	**1.37 (1.04–1.82)**	**0.028**	**1.38 (1.04–1.83)**	**0.026**
Recessive	GA+GG	314 (89.5%)	400 (92.8%)	1.00 (reference)			
	AA	37 (10.5%)	31 (7.2%)	1.52 (0.92–2.50)	0.102	1.53 (0.93–2.52)	0.098
Allele	G	474 (67.5%)	631 (73.2%)	1.00 (reference)			
	A	228 (32.5%)	231 (26.8%)	**1.31 (1.06–1.64)**	**0.014**		
rs763780							
	TT	241 (68.7%)	284 (65.7%)	1.00 (reference)			
Heterozygous	TC	100 (28.5%)	132 (30.6%)	0.89 (0.65–1.22)	0.462	0.89 (0.65–1.21)	0.459
Homozygous	CC	10 (2.8%)	16 (3.7%)	0.73 (0.33–1.65)	0.453	0.72 (0.32–1.63)	0.434
Dominant	TT	241 (68.7%)	284 (65.7%)	1.00 (reference)			
	CC+TC	110 (31.3%)	148 (34.3%)	0.87 (0.65–1.18)	0.375	0.87 (0.65–1.18)	0.369
Recessive	TC+TT	341 (97.2%)	416 (96.3%)	1.00 (reference)			
	CC	10 (2.8%)	16 (3.7%)	0.76 (0.34–1.70)	0.504	0.75 (0.34–1.58)	0.485
Allele	T	582 (82.9%)	700 (81.0%)	1.00 (reference)			
	C	120 (17.1%)	164 (19.0%)	0.88 (0.68–1.14)	0.335		

The genotyping was successful in 351 cases and 431 controls for rs2275913; The genotyping was successful in 351 cases and 432 controls for rs763780; Bold values are statistically significant (*P* < 0.05).

* Adjust for age and sex.

Stratified analyses were conducted according to sex, age, smoking, alcohol and BMI (Table 3). For rs2275913, a significantly increased CRC risk with the AA genotype was found among smokers (AA vs. GG, OR 2.38; 95% CI, 1.19–4.76; *P* = 0.014) and non-drinkers (AA vs. GG, OR 4.22; 95% CI, 1.72–9.82; *P* < 0.001). The increased effect also appeared stronger in the subgroup of age ≥ 60 years (AA vs. GG, OR 2.44; 95% CI, 1.29–4.60; P = 0.006) and females (GA vs. GG, OR 1.42; 95% CI, 1.02–1.98; *P* = 0.037). No significant findings were obtained in the analysis of rs2275913 polymorphism and BMI. However, stratified analyses by sex, age, smoking and drinking indicated that the allele or genotype frequencies of rs763780 polymorphism did not differ significantly between the cases and controls. Next, we investigated the association between these two SNPs and clinicopathologic features of CRC patients (Table 4). The AA genotype of rs2275913 polymorphism is more frequent in patients with TNM stage III+IV and in patients with lymph node metastasis. This indicated that *IL-17* rs2275913 polymorphism was correlated with TNM stage and lymph node metastasis (*P* = 0.043 and 0.026, respectively). We observed no evidence of association between rs763780 polymorphism and other clinical characteristics such as CRP, ESR, histological grade, TNM stage, tumor size, lymph node metastasis, family history and histology.

**Table 3:** Stratified analyses between rs2275913 polymorphisms and the risk of colorectal cancer

Variable	(Case/Control)			Heterozygous model	Homozygous model	Recessive model	Dominant model
rs2275913	GG	GA	AA	GA vs GG	AA vs GG	AA vs GG+GA	AA+GA vs GG
Sex							
Male	38/46	23/30	8/3	0.93 (0.46–1.86); 0.833	3.23 (0.80–13.02); 0.100	3.32 (0.85–13.06); 0.086	1.14 (0.59–2.18); 0.699
Female	122/185	131/139	29/28	**1.42 (1.02–1.98); 0.037**	1.56 (0.89–2.76); 0.124	1.32 (0.77–2.28); 0.315	**1.45 (1.05–1.98); 0.022**
Smoking							
Yes	86/118	79/88	26/15	1.23 (0.82–1.86); 0.321	**2.38 (1.19–4.76); 0.014**	**2.16 (1.11–4.22); 0.023**	1.40 (0.95–2.06); 0.091
No	74/113	75/81	11/16	1.40 (0.91–2.15); 0.124	1.04 (0.46–2.37); 0.925	0.89 (0.40–1.98); 0.776	1.34 (0.89–2.03); 0.163
Alcohol							
Yes	102/126	87/100	16/22	1.07 (0.72–1.57); 0.747	0.89 (0.45–1.79); 0.746	0.87 (0.44–1.70); 0.675	1.04 (0.71–1.50); 0.857
No	58/105	67/69	21/9	**1.76 (1.11–2.80); 0.017**	**4.22 (1.72–9.82); <0.001**	**3.24 (1.44–7.33); 0.005**	**2.04 (1.31–3.18); 0.002**
Age (years)							
<60	58/81	66/64	7/13	1.44 (0.89–2.33); 0.138	0.75 (0.28–2.00); 0.568	0.63 (0.24–1.63); 0.340	1.32 (0.83–2.11); 0.237
≥60	102/150	88/105	30/18	1.23 (0.84–1.79); 0.295	**2.44 (1.29–4.60); 0.006**	**2.23 (1.21–4.12); 0.011**	1.40 (0.98–2.00); 0.064
BMI							
<25	78/126	75/88	17/16	1.37 (0.90–2.08); 0.144	1.70 (0.81–3.57); 0.158	1.48 (0.73–3.02); 0.282	1.42 (0.95–2.11); 0.086
≥25	82/105	79/81	20/15	1.25 (0.82–1.91); 0.304	1.71 (0.82–3.54); 0.150	1.54 (0.76–3.11); 0.228	1.32 (0.88–1.98); 0.176

rs763780	TT	TC	CC	TC vs TT	CC vs TT	CC vs TT+TC	CC+TC vs TT
Sex							
Male	46/51	20/23	3/5	0.96 (0.47–1.98); 0.921	0.67 (0.15–3.00); 0.591	0.67 (0.16–2.92); 0.597	0.91 (0.46–1.80); 0.788
Female	195/233	80/109	7/11	0.87 (0.62–1.23); 0.442	0.76 (0.29–1.99); 0.573	0.79 (0.30–2.06); 0.629	0.86 (0.62–1.21); 0.388
Smoking							
Yes	133/144	52/68	7/9	0.83 (0.54–1.27); 0.391	0.84 (0.31–2.33); 0.740	0.89 (0.33–2.44); 0.823	0.83 (0.55–1.25); 0.375
No	108/140	48/64	3/7	0.97 (0.62–1.52); 0.878	0.55 (0.14–2.18); 0.397	0.56 (0.14–2.19); 0.403	0.93 (0.596–1.43); 0.726
Alcohol							
Yes	133/168	67/74	5/7	1.14 (0.76–1.70); 0.531	0.90 (0.28–2.89); 0.855	0.86 (0.27–2.75); 0.800	1.12 (0.76–1.65); 0.581
No	108/116	33/58	5/9	0.61 (0.37–1.01); 0.054	0.72 (0.25–2.08); 0.537	0.82 (0.29–2.37); 0.717	0.63 (0.39–1.01); 0.051
Age (years)							
<60	88/100	38/52	5/7	0.83 (0.50–1.38); 0.473	0.81 (0.25–2.65); 0.730	0.86 (0.27–2.78); 0.803	0.83 (0.51–1.35); 0.447
≥60	153/184	62/80	5/9	0.93 (0.62–1.38); 0.707	0.67 (0.22–2.03); 0.472	0.68 (0.22–2.06); 0.494	0.90 (0.61–1.32); 0.591
BMI							
<25	114/140	48/80	7/9	0.73 (0.47–1.13); 0.159	0.95 (0.34–2.63); 0.919	1.05 (0.38–2.88); 0.922	0.75 (0.50–1.14); 0.184
≥25	127/144	52/52	3/7	1.13 (0.72–1.78); 0.586	0.49 (0.12–1.92); 0.303	0.47 (0.12–1.84); 0.279	1.06 (0.68–1.64); 0.804

BMI, body mass index.

Bold values are statistically significant (*P* <0.05).

**Table 4:** The stratified analysis between rs2275913/rs763780 polymorphisms and clinical characteristics of CRC patients

Characteristics	Genotype distributions			
rs2275913	GG	GA	AA	GA+AA
Histological grade				
MD/WD	130/16	117/15	27/3	144/18
OR (95%CI); *P*-value	1.0 (reference)	0.96 (0.46–2.03); 0.915	1.11 (0.30–4.07); 0.877	0.99 (0.48–2.01); 0.966
Histological grade				
PD/WD	14/16	22/15	7/3	29/18
OR (95%CI); *P*-value	1.0 (reference)	1.68 (0.63–4.43); 0.296	2.67 (0.58–12.33); 0.201	1.84 (0.73–4.66); 0.195
TNM stage				
III+IV/I+II	70/90	86/68	23/14	109/82
OR (95%CI); *P*-value	1.0 (reference)	**1.63 (1.04–2.54); 0.032**	**2.11 (1.01–4.40); 0.043**	**1.71 (1.12–2.61); 0.013**
Tumor size				
>5 cm/≤5 cm	99/61	82/72	21/16	103/88
OR (95%CI); *P*-value	1.0 (reference)	0.70 (0.45–1.10); 0.122	0.81 (0.39–1.67); 0.565	0.72 (0.47–1.11); 0.133
Lymph node metastasis				
Yes/No	63/97	80/74	22/15	102/89
OR (95%CI); *P*-value	1.0 (reference)	**1.67 (1.06–2.61); 0.025**	**2.26 (1.09–4.68); 0.026**	**1.77 (1.15–2.70); 0.009**
Family history				
Yes/No	24/136	17/137	6/31	23/168
OR (95%CI); *P*-value	1.0 (reference)	0.70 (0.36–1.37); 0.298	1.10 (0.41–2.91); 0.853	0.78 (0.42–1.44); 0.418
Histology				
Adenocarcinoma/Not	155/5	149/5	33/4	182/9
OR (95%CI); *P*-value	1.0 (reference)	0.96 (0.27–3.39); 0.951	0.27 (0.07–1.05); 0.058	0.65 (0.21–1.99); 0.452
ESR				
≥10/<10	63/97	68/86	17/20	85/106
OR (95%CI); *P*-value	1.0 (reference)	1.22 (0.78–1.91); 0.391	1.31 (0.64–2.69); 0.464	1.24 (0.81–1.89); 0.333
CRP				
≥25/<25	13/147	9/145	7/30	16/175
OR (95%CI); *P*-value	1.0 (reference)	0.70 (0.29–1.69); 0.431	2.64 (0.97–7.17); 0.057	1.03 (0.48–2.22); 0.932

rs763780	TT	TC	CC	TC+CC
Histological grade				
MD/WD	187/23	77/11	9/1	87/11
OR (95%CI); *P*-value	1.0 (reference)	0.86 (0.40–1.85); 0.701	0.49 (0.10–2.46); 0.378	0.97 (0.45–2.09); 0.943
Histological grade				
PD/WD	31/23	12/11	0/0	12/11
OR (95%CI); *P*-value	1.0 (reference)	0.81 (0.30–2.16); 0.672	NA	0.81 (0.30–2.16); 0.672
TNM stage				
III+IV/I+II	116/125	39/61	5/5	44/66
OR (95%CI); *P*-value	1.0 (reference)	0.69 (0.43–1.11); 0.123	1.08 (0.30–3.82); 0.908	0.72 (0.46–1.14); 0.156
Tumor size				
>5 cm/≤5 cm	135/106	60/40	7/3	67/43
OR (95%CI); *P*-value	1.0 (reference)	1.18 (0.73–1.89); 0.499	1.83 (0.46–7.26); 0.382	1.22 (0.77–1.94); 0.390
Lymph node metastasis				
Yes/No	86/155	31/69	5/5	36/74
OR (95%CI); *P*-value	1.0 (reference)	0.81 (0.49–1.33); 0.407	1.80 (0.51–6.40); 0.356	0.88 (0.54–1.41); 0.589
Family history				
Yes/No	32/209	15/85	1/9	15/95
OR (95%CI); *P*-value	1.0 (reference)	1.15 (0.59–2.24); 0.675	0.73 (0.09–5.92); 0.764	1.03 (0.53–1.99); 0.927
Histology				
Adenocarcinoma/Not	231/10	96/4	9/1	105/5
OR (95%CI); *P*-value	1.0 (reference)	1.04 (0.32–3.39); 0.950	0.39 (0.05–3.38); 0.393	0.91 (0.30–2.73); 0.865
ESR				
≥10/<10	100/141	37/63	2/8	39/71
OR (95%CI); *P*-value	1.0 (reference)	0.83 (0.51–1.34); 0.441	0.35 (0.07–1.70); 0.193	0.78 (0.49–1.24); 0.284
CRP				
≥25/<25	12/229	6/94	1/9	7/103
OR (95%CI); *P*-value	1.0 (reference)	1.22 (0.44–3.34); 0.802	2.12 (0.25–18.13); 0.492	1.30 (0.50–3.39); 0.596

Bold values are statistically significant (*P* < 0.05). PD, poorly differentiation, MD, moderately differentiation, WD, well differentiation; TNM, tumor node metastasis.

## Discussion

In the present study, we found that *IL-17A* rs2275913 polymorphism was associated with the increased risk for CRC in a Chinese population. However, no association was observed for the *IL-17F* rs763780 polymorphism. Additionally, rs2275913 polymorphism showed significant correlation with TNM stage and lymph node metastasis in CRC patients.

Recently, several studies investigated the association between *IL-17A* rs2275913 polymorphism and risk of CRC [9,11,13–15]. Omrane et al. firstly conducted a population-based study to explore the association of rs2275913 polymorphism with CRC risk in a Tunisian population involving 102 CRC patients and 139 controls [11]. They found that *IL-17A* rs2275913 polymorphism conferred susceptibility to CRC and was associated with tumor location and tumor differentiation [11]. However, no significant association with CRC risk was observed for rs2275913 polymorphism in another Tunisian study conducted by Bedoui et al. [15]. Additionally, two Caucasian studies also investigated the effect of this SNP on the risk of CRC [9,13]. Nemati et al. revealed that AG genotype showed a significantly elevated risk for CRC compared to GG genotype in the Iranian population [13]. This significant association was also observed in the Saudi population [9]. However, no study has explored this SNP in the Chinese population. In this study, we observed that *IL-17A* rs2275913 polymorphism increased the risk of CRC under the additive, dominant and allelic models. The significant association observed in the whole population was also shown in the female, smoker, drinker and individuals with age ≥ 60 years groups. According to the dbSNP database, rs2275913 polymorphism was located in the promoter region of *IL-17A* gene. We hypothesized that *IL-17A* rs2275913 polymorphism conferred susceptibility to CRC by altering the IL-17 expression. In addition, *IL-17A* rs2275913 polymorphism was related to TNM stage III+IV and lymph node metastasis.

*IL-17F* rs763780 polymorphism was also investigated in several studies [9,10,12–14]. Three studies failed to find allele or genotype association with CRC susceptibility [9,10,14]. However, Ma et al. found that CC genotype or C allele was associated with the increased risk of CRC in a Chinese population [12]. Significant associations with the CRC risk were also demonstrated in an Iranian population [13]. In the present study, there was no significant association for this SNP in the overall analysis and in the stratified analyses of sex, age and smoking. Obviously, the findings of this study were consistent with most previous studies [9,10,14]. The rs763780 polymorphism is a missense mutation (His > Arg) when the nucleotide changes from T to C. Key amino acid changes may affect the three-dimensional structure of the protein, which in turn leads to changes in protein function. DbSNP database indicated that this mutation was benign. This may be the reason why we failed to obtain the positive results.

Several limitations of the present study need to be addressed. First, the sample size was not large, thus we could not rule out the possibility of false-positive results. Second, environmental factors might have affected the final results, including occupation and educational level. Third, we did not follow up on CRC patients, limiting our further analysis. Fourth, the controls from the hospital may not fully represent the entire population.

In conclusion, *IL-17A* rs2275913 polymorphism is associated with increased risk for CRC in a Chinese population. Furthermore, this SNP was associated with higher TNM stage and regional lymph node metastasis. However, no positive findings were obtained for *IL-17F* rs763780 polymorphism. Further studies in other studies with larger sample sizes, as well as functional evaluation of studied SNPs, are warranted to further validate these findings.

## Abbreviations

CRC: colorectal cancer
CRP: C-reactive protein
IL: interleukin
SNP: single-nucleotide polymorphism
